# Identification of similar regions of protein structures using integrated sequence and structure analysis tools

**DOI:** 10.1186/1472-6807-6-4

**Published:** 2006-03-09

**Authors:** Brandon Peters, Charles Moad, Eunseog Youn, Kris Buffington, Randy Heiland, Sean Mooney

**Affiliations:** 1 Center for Computational Biology and Bioinformatics, Indiana University School of Medicine, Indianapolis, IN 46202, USA; 2 Scientific Data Analysis Lab, Pervasive Technology Labs, Indiana University, Indianapolis, IN 46202, USA

## Abstract

**Background:**

Understanding protein function from its structure is a challenging problem. Sequence based approaches for finding homology have broad use for annotation of both structure and function. 3D structural information of protein domains and their interactions provide a complementary view to structure function relationships to sequence information. We have developed a web site  and an API of web services that enables users to submit protein structures and identify statistically significant neighbors and the underlying structural environments that make that match using a suite of sequence and structure analysis tools. To do this, we have integrated S-BLEST, PSI-BLAST and HMMer based superfamily predictions to give a unique integrated view to prediction of SCOP superfamilies, EC number, and GO term, as well as identification of the protein structural environments that are associated with that prediction. Additionally, we have extended UCSF Chimera and PyMOL to support our web services, so that users can characterize their own proteins of interest.

**Results:**

Users are able to submit their own queries or use a structure already in the PDB. Currently the databases that a user can query include the popular structural datasets ASTRAL 40 v1.69, ASTRAL 95 v1.69, CLUSTER50, CLUSTER70 and CLUSTER90 and PDBSELECT25. The results can be downloaded directly from the site and include function prediction, analysis of the most conserved environments and automated annotation of query proteins. These results reflect both the hits found with PSI-BLAST, HMMer and with S-BLEST. We have evaluated how well annotation transfer can be performed on SCOP ID's, Gene Ontology (GO) ID's and EC Numbers. The method is very efficient and totally automated, generally taking around fifteen minutes for a 400 residue protein.

**Conclusion:**

With structural genomics initiatives determining structures with little, if any, functional characterization, development of protein structure and function analysis tools are a necessary endeavor. We have developed a useful application towards a solution to this problem using common structural and sequence based analysis tools. These approaches are able to find statistically significant environments in a database of protein structure, and the method is able to quantify how closely associated each environment is to a predicted functional annotation.

## Background

Automated functional annotation of proteins based on their sequence and structure is a challenging and important problem [1]. One area of interest to us is the identification of regions in protein structures that are statistically associated with a given structural or functional annotation. To provide a useful resource addressing this problem, we have developed web tools for identification of sequence conserved residues and environments structurally associated with specific functional and structural annotations.

Projects such as Structural Classification of Proteins (SCOP) [2] or CATH [3] annotate the known protein structure universe heirarchically. For example, SCOP classifies protein by class, fold, superfamily and family. While these annotations often cluster into groups that represent function, some functional annotations do not transfer well across shared structural similarity. To annotate function, typically enzyme classification numbers [4] (EC, for enzymes) and/or gene ontology (GO) [5] codes are used. EC numbers are heirarchical and are built as a mechanism to annotate and classify overall enzyme chemistry. GO is a more recent project aimed at developing an ontology for annotation of molecular function, biological process and cellular component.

Sequence based approaches have evolved to become better at identifying distant homologs. Initially, BLAST [6] was commonly used to perform structural and functional annotation transfer. Profile based approaches such as PSI-BLAST [7] and Hidden Markov Models (HMMs) using HMMer  are generally preferred over BLAST for improved remote homolog detection [1]. HMMs can be built from gold standard alignments to search for distant homology in a supervised way [8]. For example, the SUPERFAMILY model dataset contains SUPERFAMILY HMM models built for use with the HMMer software [9].

Similarly, structural approaches have traditionally relied on structural superpositions to identify structural similarity. These tools include Dali [10], Combinatorial Extension (CE) [11] or MinRMS [12]. Other unsupervised methods that find structural neighbors include tools such as VAST [13], the method of Singh and Saha [14], PINTS [15], and LFF [16]. More recent methods such as the Match Augmentation Algorithm, relies on an evolutionary trace approach [17] to define a template that can be searched from within a database [18]. As a complementary addition to these and other methods, we have developed the Structure-Based Local Environment Search Tool (S-BLEST) as an unsupervised approach for discovering structurally conserved environments within protein structures [19]. S-BLEST is based on the FEATURE [20] representation of a local structural environment, and rapidly searches databases of vectors of local structure properties using nearest neighbor queries. These matched environments can be used in several ways. First, S-BLEST can combine different residue environment queries from a single protein using a congruence algorithm to find structurally similar proteins in a database, and the environments that confer that similarity. Second, the environment can be associated with a structural or functional annotation by determining how well the other proteins that are annotated with a specific annotation are highly ranked in the query results. This can be quantified using the area under a receiver operator characteristics (ROC) curve.

The philosophy and/or the methods of the previously described approaches have been used to develop resources for the prediction of function from uncharacterized proteins. The DBAli tools provide CATH, SCOP, EC, GO and keyword annotations for a protein structure [21]. ProFunc uses sequence, structure and residue templates to characterize proteins of interest [22]. ProKnow is a resource for annotating GO terms using Bayes' theorem and protein structure [23]. WebFeature uses supervised learning to train models of protein environments for inferring functional sites in protein structures [24].

Here, we have integrated S-BLEST, PSI-BLAST and HMMer to report sequence and structurally similar regions of protein domains. We then use S-BLEST to estimate structural residue environment conservation and PSI-BLAST to estimate sequence conservation. In total we have built an automated pipeline for analysis of PDB formatted coordinate data, a website for analysis of the results and a suite of web services for extending tools to access these methods. We have further extended UCSF Chimera [25] and Delano Scientific PyMOL  to use our web services.

### Implementation

The underlying analysis methods are based on PSI-BLAST [7], HMMer , and S-BLEST [19]. Here we have created an intuitive interface based on both a web site and an authenticated web services API. We then extended commonly used applications for protein structure analysis to take advantage of our services.

#### Method overview

When structural coordinates are submitted to our service, the structural coordinates are submitted to S-BLEST and the sequence is submitted to PSI-BLAST and HMMer using the following approaches.

For PSI-BLAST, the sequence is queried against the database specified by the user upon submission. Usually, we recommend using ASTRAL 40 v1.69 [26]. PSI-BLAST (blastpgp) is run on our servers for three iterations. All output files are stored in a private job directory that is shared with the other methods, and all output options are available to the submitter. Additionally, the degree of conservation across the submitted sequence is determined using the position specific scoring matrix (PSSM) output from the blastpgp program.

After the PSI-BLAST job is initiated, HMMer is run against the SUPERFAMILY library of HMM models [9]. Each statistically significant hit with e-value less than 10e-10 is determined, and the SCOP superfamily is tabulated. After running against the more than 10,000 models, the top superfamilies are determined, and the top e-value to a specific model is reported. Note that there are often multiple models for each superfamily, only the top e-value is reported.

The S-BLEST job takes several steps. First, to perform a query, a residue environment is encoded as a vector of properties using a procedure similar to others [15,16]. To describe the local environment for each residue, a vector of atom-based properties is determined from four 1.875 Å concentric shells extending outward from the position of the residue's beta-carbon atom (Cβ). In the case of glycine residues, the vector is centered in a position where a Cβ would lie. This is determined using the procedure described previously [19]. The list of properties is available from the authors upon request, and are normalized based on the minimum and maximum values of each property in the database being queried. The vectors from the specified database of protein structures are then used to search against using Manhattan distance for determining vector similarity. Each residue environment is queried against the database, and all environments with a Z-score of better than -2.5 are tabulated. The results for each residue are stored in a file with the following naming format: "USER.<*residue number*>.<*chain*>.<*insertion code*>", where spaces (empty chains and insertion codes) are replaced with underscore characters and the 'USER' represents a user submitted structure (internally, we support PDB ID's or ASTRAL domain ID's in place of 'USER' and this may be implemented on the public website in the future). These files are colloquially referred to as USER files. Once all residues are queried, the protein domains are identified by ranking the average top Z-scores from the specified number of best residues from each domain. Then, a congruence algorithm [27] is performed that combines the USER files by finding the best subset of the user specified number of residues to rank the protein chains in the database relative to the query.

Once PSI-BLAST, HMMer and S-BLEST are completed, the proteins containing either PSI-BLAST high scoring segment pairs (hsps) of better than 10e-10 significance or S-BLEST Z-scores less than the user submitted value (our parameterized default is -5.4) are ranked and reported. From those hits, the common SCOP [2] family, SCOP superfamily, GO terms [5] and EC numbers [4] are collected. If a HMMer predicted SCOP superfamily is not common with these hits, it is added to the list. When a user clicks on the "prediction of function summary" link on the results page, the structural environments and sequence residues most associated with these annotations can be determined. For S-BLEST, this is determined by calculating the area under an ROC plot for each USER file, by setting the residue environments as "+" if it is in a protein domain annotated with the query annotation (SCOP family, superfamily, etc.) and "-" if it is not in a protein domain containing that annotation. By applying to each USER file, the structurally conserved and unique residue environments most associated with an annotation is determined. This is plotted on the "prediction of function summary page." Additionally, the most conserved PSI-BLAST residues are plotted similarly using the relative conservation value reported in the PSSM output (first column after the individual amino acid scores).

The user has the ability to select the dataset to search against. We currently provide nonredundant sets of protein structures and domains. The ASTRAL Compendium provides PDB style coordinates of domains annotated with SCOP IDs and with maximum redundancy at 40, 95 or 100% sequence identity. Furthermore, the PDB [28] provides clusters of structures based on 50% and 70% sequence identity. We have selected the first structure from each cluster to create a searchable dataset. The default is ASTRAL 40 v1.69, and that usually represents sufficient coverage of the protein domain universe for detection.

#### Coordinate submission

When submitting coordinate data from the S-BLEST website, the user uploads a PDB formatted file and specifies the protein chain to be analyzed. The user also enters an email address, the minimum Z-score, the number of residue environments to match, and the database to query against. Upon submission, the coordinates are stored on the server and a job ID is generated. The submission is then run on our network and the output files are generated. An email is then sent to the user indicating that their results are ready and provides a link to the results page for the job.

#### Web site

The website portion of S-BLEST is built using several scripts written in PHP and Python. The underlying job management is stored in a MySQL database. The vector encoding and database searching is performed using the S-BLEST software, developed in C.

#### Web services

As an alternative to the website interface of S-BLEST, we provide web services that fully encompass the features as described above. Implementing structural data mining tools such as those described above in a web service is attractive because they allow for easy development of software that interacts with the underlying methods and they allow for integration of data from multiple sources. Additionally, content providers are able to maintain their own datasets and tools, ensuring that researchers are always up to date. Here, we have developed both a traditional web site and an API to the method using the SOAP protocol. With these tools, users can interactively analyze structurally conserved regions in query protein structures and assess their statistical significance. Furthermore, residue environments that are associated with a particular function or structural annotation can be identified and quantified.

Methods are provided to allow remote programs to submit structures, manage jobs, and retrieve results. We also provide a suite of protein structure related services that complement S-BLEST. Developers can utilize these methods for use in interactive applications or batch processing jobs. Web services do not bind a developer to a specific programming language, so they provide a flexible alternative to the standard web interaction. Our services provide authenticated access to our protein structure analysis tools, structurally similar environments to queries and function prediction of specific residue environments.

#### Plugin extensions

Client plug-ins to two widely used protein visualization applications, UCSF's Chimera [25] and Delano Scientific's PyMOL , were developed using the Python programming language. We developed a web service container and server using a feature rich networking toolkit, Twisted . Using this library, we serve data and methods through the web service transport, SOAP. All the accessible services are dynamically documented and self-described in the standard web service Description Language (WSDL) format at the Lifescience web site . Both of these applications provide extensive developer API's which we utilize in order to map the data from the web services to protein structure. Nearly all features of the website are accessible using the plug-ins. Initially, after a job is reported as complete, the best hits are summarized in a pull down menu. Each residue environment that has a significant match (Z-score) to that hit, is reported in the text box below the hits pull down menu. Selection of environments in the text box selects them on the structure, and performs a superposition of the two structures using the backbone atoms of the selected residues. When users click on the 'Function' tab, all of the structural and functional annotations reported on the website are reported and the area under the ROC plots are ranked [19]. In Chimera, clicking the 'Plot' button pops up a user interactive plot of the scores that selects residues on the structure based on the user clicked minimum threshold. Additionally, a link is provided in the plug-in window that opens a web browser with the corresponding webpage for that query.

### Interface features

#### The S-BLEST website

**Figure 1:**
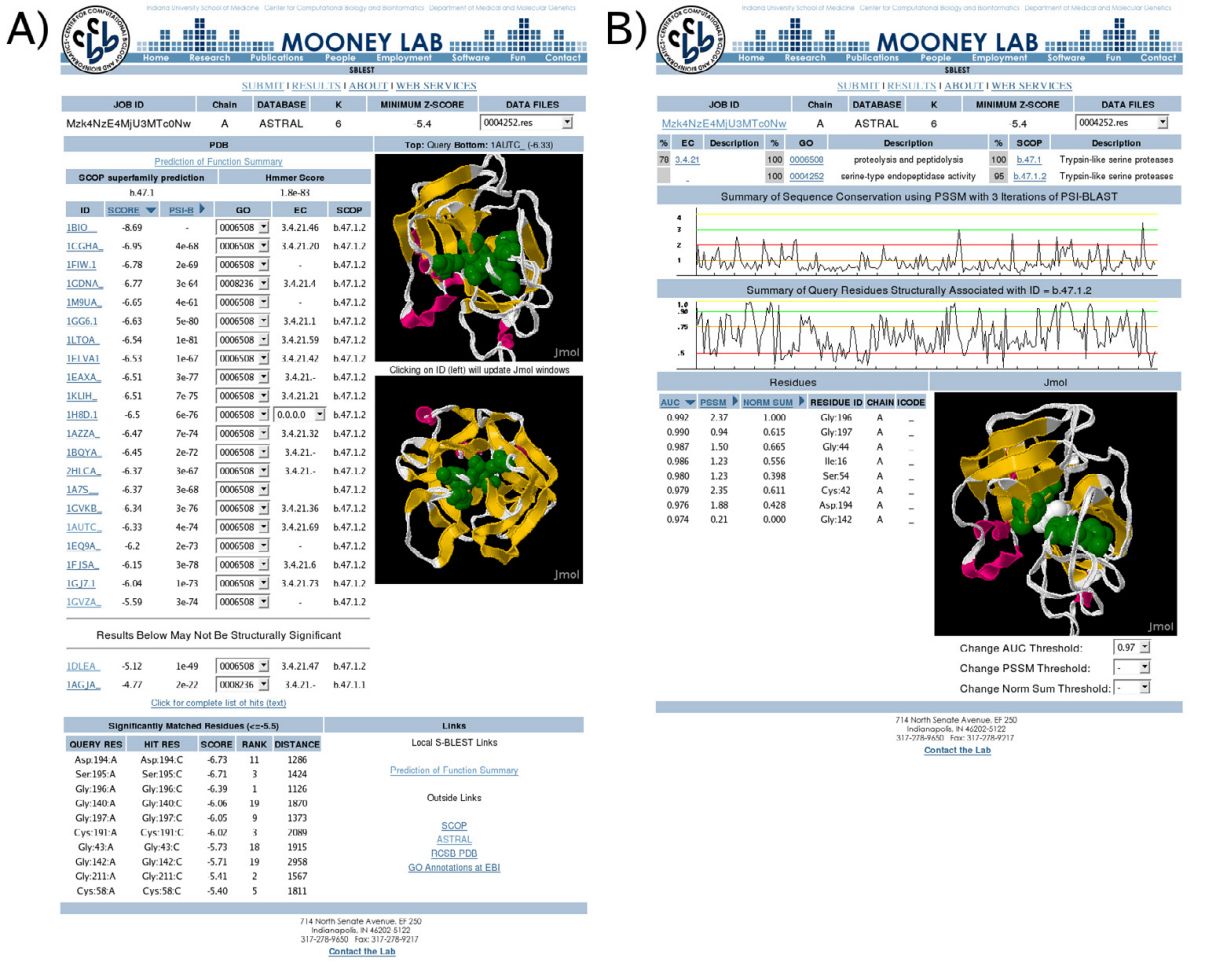
**Example of results as viewed in the S-BLEST website**. A) The results of a serine protease (PDB:1DSUA) against ASTRAL 40 v1.65, showing the list of hits and the JMOL windows illustrating both the query and the selected hit. For each hit, the Z-score, the PSI-BLAST e-value, the SCOP ID, the GO annotations and the EC number are displayed, if available. B) The function prediction page of 1DSU:A, showing how close each residue environment is to the annotation of the SCOP family b.47.1.2, trypsin fold serine proteases and how conserved the residues are in the PSI-BLAST PSSM.

The S-BLEST website provides an interface to submit jobs and view results. Upon submission to the S-BLEST queue it takes between five and twenty minutes for a single protein of average length, depending on the size of the database being queried against. An example of a serine protease query is shown in Figure 1. When a user visits a results page, the user will see the summary information and the HMMer predicted SCOP superfamily and a link to predicted functions. Below this summary are the hits. Navigation is possible by browsing through the list of hits that have been returned in the S-BLEST/PSI-BLAST job. Each hit corresponds to a PDB ID, and is annotated with a Z-Score, a PSI-BLAST e-value, GO annotations, EC annotations, and SCOP annotations. The hits can be sorted by PSI-BLAST e-value or S-BLEST Z-score. Each hit link takes the user to a results page that corresponds to the selected hit. The results page for each hit contains a JMol  window for both the query PDB and the hit PDB. Also provided on this page is a list of structurally significantly matched residues and several other links to other databases (Figure 1A).

If there are significant hits, a link to the "function prediction" page will appear (Figure 1B). Clicking on this link will forward the user to a page that identifies the common SCOP, EC and GO annotations of the hits, and displays the percentage of hits that share that annotation. Below the annotations are two plots. The first plot is the conservation reported in the PSI-BLAST PSSM output file. The second plot displays the residue environments structurally associated with the annotation (AUC of an ROC, see [19]). Clicking on a prediction updates the second plot to correspond to that specific annotation. Below the plots, the structure is displayed in a JMol window with a quantification of which residues are high scoring. Users can view sequence conservation, structural conservation or a normalized sum combination of the two; additionally thresholds can be added that limit the display of highlighted residues in the JMol window.

#### Plugin extensions

Using the supported applications (UCSF Chimera and PyMOL), a user can interactively submit protein structure data to our S-BLEST tools to be processed. All jobs are managed by the server and a user can view the job history by displaying parameters and metadata associated with a specific query and by checking the completion status. When a job has completed, the user can view the top hits, as determined by S-BLEST, and choose to perform structural alignments between the submitted structure and a statistically significant hit.

**Figure 2:**
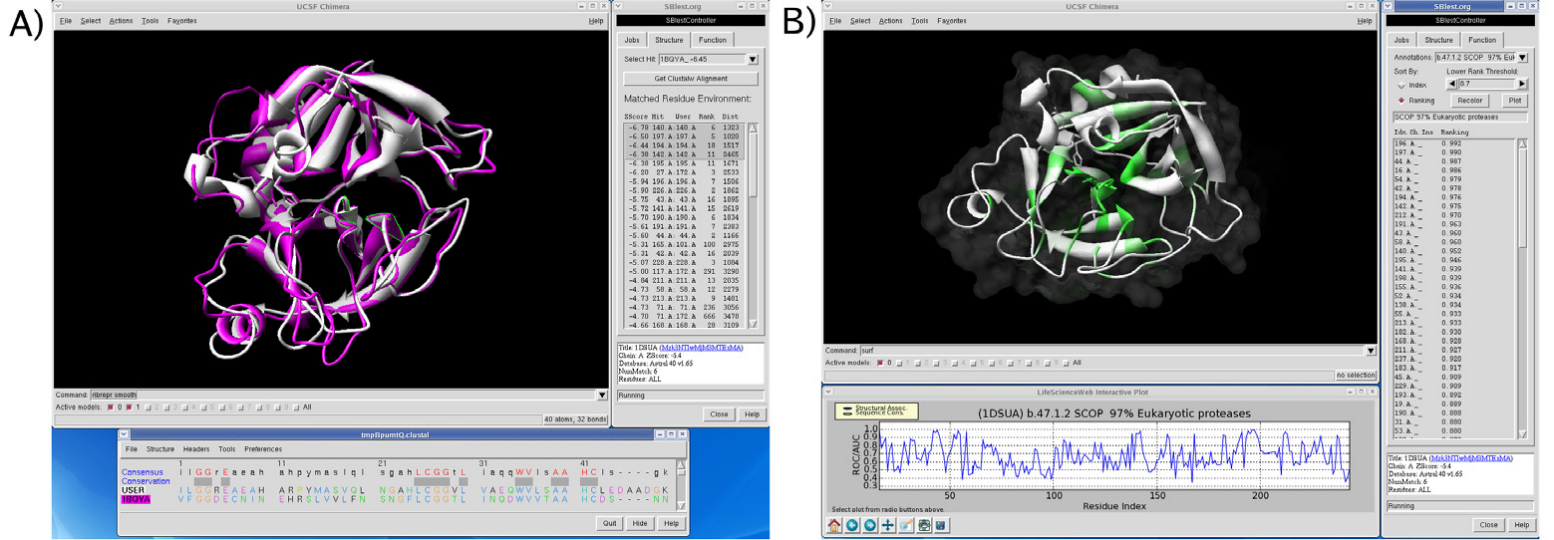
**Example of the interactive plugins developed for UCSF Chimera using the serine protease example from Figure 1**. A) Each hit is displayed in a pull down menu with the associated Z-score. When selected from the menu, the structure is downloaded from our web service and displayed alongside the query. A text box in the plug in window displays all matched environments between the query and the hit and the associated Z-score. When those environments are individually selected, the query and the hit are superimposed using the Chimera match command based on the backbone atoms of the matched residues. A ClustalW based alignment is available by clicking the appropriate button. B) The common functional annotations of the hits are used to annotate the query structure by determining how closely associated each environment is to a given annotation. The most closely associated residues are highlighted in darker shades of green, and different thresholds can be selected.

The function annotation results can also be mapped to the submitted structure. The user selects the SCOP, GO, or EC annotation of interest, and the plug-in will map the AUC values on the structure through a color gradient representing the AUC magnitude. Additionally, Chimera users are able to plot the results using the Matplotlib library and select regions of the plot to view on the protein structure. Both PSSM and AUC plots are available for display. This visual representation of the function annotations allow for a user to quickly hypothesize what environments are most likely associated with the functional site of a protein. An example of both the protein results and the function analysis of the plug-in are shown in Figure 2.

## Results

### Evaluation and limitations of the method

**Figure 3:**
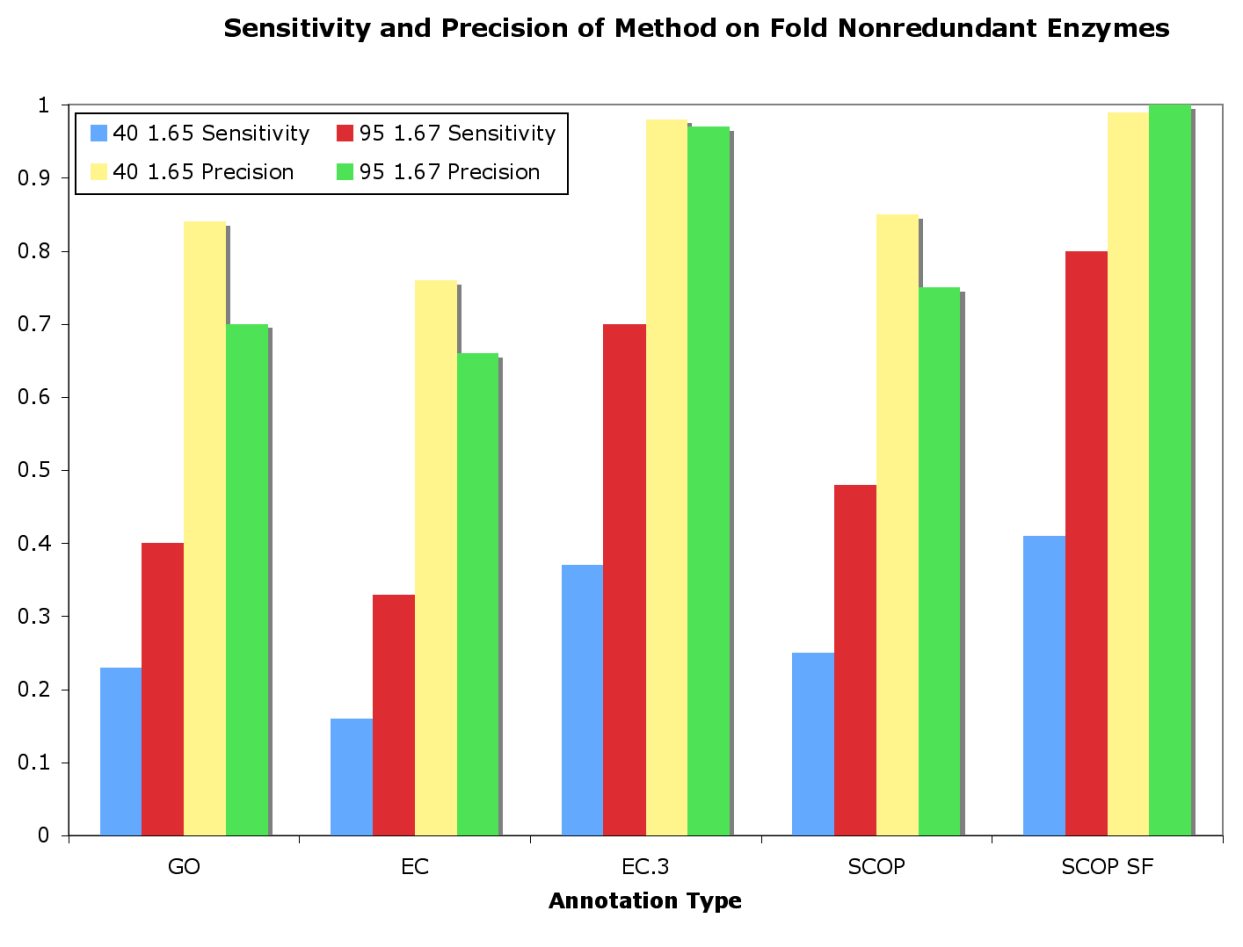
**Average performance of the method on a fold nonredundant enzyme dataset**. All folds in ASTRAL 40 v1.65 with annotations in the Catalytic Site Atlas (CSA) were collected, and from each a random member was selected. Then each representative was run against the method using the database specified in the legend, with the HMMer results excluded. The same set was run against ASTRAL 95 v1.67, if the domain was not found in v1.67, another random member of that fold was chosen, if the fold was not found in v1.67, it was excluded. The sensitivity and precision were measured as (true positives/total positives) and (true positives/total hits), respectively. EC.3 is the first three numbers of the EC number and SCOP SF is the first three numbers of the SCOP ID (superfamily).

To evaluate the method, we determined the real world performance of annotation transfer on enzymes. This was performed by determining the sensitivity and precision of the method when predicting SCOP family, Superfamily, GO term and EC number on a random member of each enzyme fold in ASTRAL 40 v1.65. Each protein was run against the method, and since it is contained in the database being queried it was removed from both the PSI-BLAST and S-BLEST results to prevent incorrect accuracies. HMMer superfamily predictions were not included, as the models were likely trained with the query protein. Additionally, all domains spanning multiple chains were not included, since PSI-BLAST results from multiple chains are difficult to analyze and the website currently only supports analysis of single chains. We applied this to ASTRAL 40 v1.65 and ASTRAL 95 v1.67 to evaluate how the method performs on homologs with less than 40% identity and on a newer dataset with many similar homologs. Not surprisingly, including similar sequences from a newer dataset improves the results and the sensitivity, dramatically (Figure 3). Sensitivity is low, in order to keep precision as high as possible. If the user wants to increase sensitivity, the threshold can be lowered upon submission and precision will be reduced, perhaps significantly. The difficulties in functional annotation transfer can be seen in the figure. Clearly, the method performs well on SCOP superfamily prediction and the first three numbers of the EC number. GO, SCOP and EC have relatively low sensitivities (<0.5) and precision values of at least 0.7. Sensitivities should be improved by either improved remote homolog detection, more diverse libraries of proteins/domains, or more quantitative selection of annotations from the selection of hits.

We believe that the value of this method lies in identifying structural and functional annotations from statistically significant neighbors and in identifying residues and structural environments that are associated with those annotations. There exist structural environments that are conserved with little sequence similarity and vice versa. As a remote homolog detection tool, this resource will only find more hits than PSI-BLAST if there are highly conserved structural environments between the query and the hit. This does occur, for example ASTRAL domain d12asa_ (asparagine synthetase) finds several significant environments in ASTRAL 40 v1.65. These environments are in d1b8aa2 (aspartyl-tRNA synthetase) with Z-score of -5.8 and in d1g51a3 (aspartyl-tRNA synthetase) with Z-score of -5.5 while only d1b8aa2 is detected with PSI-BLAST, with insignificant e-value of 0.17.

## Conclusion

Automated functional annotation of proteins is an important problem for computational biology. We have developed a resource that can quickly determine if a protein has close structural neighbors and can associate regions of that protein to the functional annotations of those neighbors. Our website accepts requests to analyze coordinates that have not been previously characterized and will identify conserved environments and make predictions when statistical significance exists. To make this useful broadly, we have extended common applications to use our computing servers to provide analysis with our method, and we encourage other researchers to extend applications using our web services framework.

## Availability and requirements

Project name: S-BLEST

Project home page: 

Operating system(s): Platform independent

Programming language: Python (for client extensions)

Other requirements: PyMOL or UCSF Chimera

License: Indiana University RTC software license

Any restrictions to use by non-academics: license required

## Authors' contributions

BP developed the underlying S-BLEST code, and the web site. CM developed the web services, the plug-in clients and aided in development of the underlying S-BLEST queuing system. EY partially developed the S-BLEST method and code. KB partially developed the S-BLEST web site and queuing system. RH advised CM and aided in design and development of the web services and client plug-ins. SDM developed the S-BLEST method, and aided in development of the S-BLEST code, web site, web services and queuing system.
